# Metagenomic sequencing of cyanobacterial-dominated Lake Victoria—an African Great Lake

**DOI:** 10.1128/mra.00798-24

**Published:** 2024-11-12

**Authors:** Brittany N. Zepernick, Lauren N. Hart, Kaela E. Natwora, Katelyn M. Brown, Julia A. Obuya, Mark Olokotum, Eric O. Okech, Natalie G. Keating, Davide Lomeo, Emma J. Tebbs, NSF-IRES 2022-2023 Lake Victoria Research Consortium, Cody S. Sheik, David H. Sherman, Gregory J. Dick, Steven W. Wilhelm, Ken G. Drouillard, Theodore Lawrence, Albert Getabu, Bethwell Owuor, Anakalo Shitandi, Reuben Omondi, James Njiru, Lewis Sitoki, Kefa M. Otiso, R. Michael L. McKay, George S. Bullerjahn

**Affiliations:** 1 Department of Microbiology, University of Tennessee Knoxville, Knoxville, Tennesse, USA; 2 NSF-IRES Lake Victoria Research Consortium, Duluth, USA; 3 Program in Chemical Biology, University of Michigan, Ann Arbor, Michigan, USA; 4 Life Sciences Institute, University of Michigan, Michigan, USA; 5 Large Lakes Observatory, University of Minnesota Duluth, Duluth, Minnesota, USA; 6 Department of Biological Sciences, Bowling Green State University, Bowling Green, Ohio, USA; 7 Kenya Marine and Fisheries Research Institute, Kisumu, Kenya; 8 National Fisheries Resources Research Institute (NaFIRRI), Jinja, Uganda; 9 Egerton University, Njoro, Kenya; 10 Department of Geography, King’s College London, London, United Kingdom; 11 Cooperative Institute for Great Lakes Research (CIGLR), University of Michigan, Ann Arbor, Michigan, USA; 12 Department of Earth and Environmental Sciences, University of Michigan, Ann Arbor, Michigan, USA; 13 Great Lakes Center for Fresh Waters and Human Health, Department of Biological Sciences, Bowling Green State University, Bowling Green, Ohio, USA; 14 Great Lakes Institute for Environmental Research, University of Windsor, Windsor, Ontario, Canada; 15 African Center for Aquatic Research and Education, Ann Arbor, Michigan, USA; 16 Kisii University, Kisii, Kenya; 17 Technical University of Kenya, Nairobi, Kenya; 18 School of Earth, Environment and Society, Bowling Green State University, Bowling Green, Ohio, USA; Indiana University Bloomington, Bloomington, Indiana, USA; 1 NSF-IRES Lake Victoria Research Consortium, National Science Foundation, USA; 2 Kenya Marine and Fisheries Research Institute, Kisumu, Kenya; 3 Kisii University, Kisii, Kenya; 4 Jaramogi Oginga Odinga University of Science and Technology, Bondo, Kenya; 5 University of Wisconsin–Milwaukee, Milwaukee, Wisconsin, USA; 6 Florida Gulf Coast University, Fort Myers, Florida, USA; 7 Department of Biological Sciences, Bowling Green State University, Bowling Green, Ohio, USA; 8 Department of Biology, Baylor University, Waco, Texas, USA; 9 University of Wisconsin–Madison, Madison, Wisconsin, USA; 10 Department of Geography, King’s College London, London, United Kingdom; 11 Aquatic Taxonomy Specialists, Malinta, Ohio, USA; 12 Arizona State University, Tempe, Arizona, USA; 13 National Fisheries Resources Research Institute (NaFIRRI), Jinja, Uganda; 14 George Mason University, Fairfax, Virginia, USA; 15 Maasai Mara University, Narok, Kenya; 16 Technical University of Kenya, Nairobi, Kenya; 17 Department of Medical and Applied Sciences, Sigalagala National Polytechnic Kakamega, Kakamega, Kenya; 18 Fort LeBoeuf School District, Erie, Pennsylvania, USA; 19 Kent State University, Kent, Ohio, USA; 20 Technical University of Mombasa, Kenya, Bondo, Kenya; 21 Department of Biological Sciences, Bowling Green State University, Bowling Green, Ohio, USA; 22 Department of Biological Sciences, Great Lakes Center for Fresh Waters and Human Health, Bowling Green State University, OH, USA, Bowling Green, Ohio, USA; 23 Great Lakes Institute for Environmental Research, University of Windsor, Windsor, Ontario, USA

**Keywords:** harmful algal blooms, Winam Gulf, cyanotoxins, climate change, time series

## Abstract

We report 40 metagenomic libraries collected from the Winam Gulf of Lake Victoria during May–July of 2022–2023 and an additional eight opportunistic libraries from adjacent Lakes Simbi, Naivasha, and regional river systems. The sampling period captured cyanobacterial bloom events – shedding insight onto community composition and genomic potential.

## ANNOUNCEMENT

Compared with North America’s Laurentian Great Lakes, the African Great Lakes have received little attention (1). This is a critical oversight given the African Great Lakes contain ~25% of all freshwater and provide potable water and food to >62 million residents (2). Lake Victoria (Uganda, Tanzania, and Kenya) has been influenced by intense agricultural and anthropogenic development. Eutrophication has spurred year-round, toxigenic cyanobacterial blooms in the Winam Gulf (Kenya), a shallow embayment of the lake (3, 4). Despite these blooms, few molecular studies have characterized the planktonic community of Lake Victoria and the broader watershed. This research gap hinders monitoring and mitigation efforts while jeopardizing the safety of residents, many of whom rely on untreated lake water for drinking (4). Here, we report metagenomic libraries collected from Lake Victoria throughout 2022 (*n* = 22) and 2023 (*n* = 18) in addition to opportunistic samples from Lakes Simbi, Naivasha and riverine systems (*n* = 8) to address this gap.

**Fig 1:**
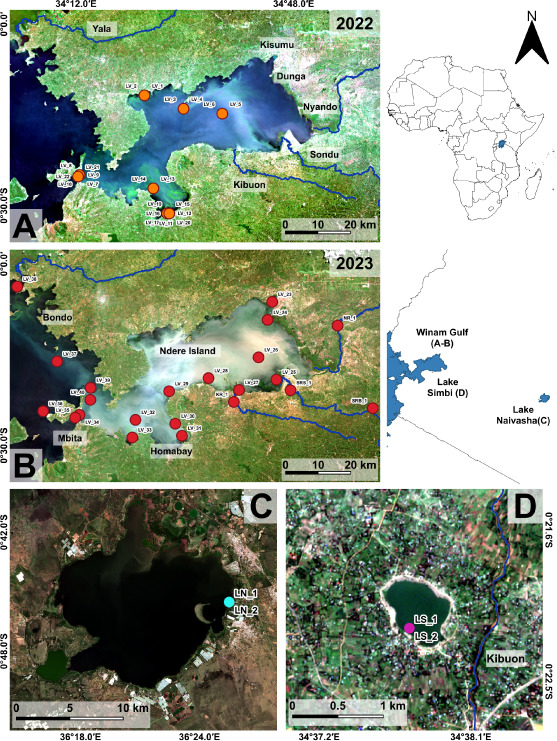
Geographic distribution of the 2022 and 2023 sample sites corresponding to the 48 metagenomic libraries generated during the NSF-IRES Advanced Studies Institute on Water Quality and Harmful Algal Blooms in Lake Victoria, Kenya. (A and B) Winam Gulf (Lake Victoria) on 30 June 2022 and 26 May 2023, respectively. Green coloration indicates algal communities (Chl *a*), and brown coloration indicates sediment plumes. (C) Lake Naivasha on 13 May 2022. (D) Lake Simbi on 26 May 2023. All images are presented as true color composites and were captured by Sentinel-2 MSI-A. We note satellite images were selected based on the absence of cloud cover and thus do not always coincide with the exact sample date. Biological replicate IDs in panel A have been consolidated to only show one replicate due to the superimposition of the label (where needed with LV_17 and LV_18). True color images composed by Sentinel-2. Images were compiled together via QGIS LTR (v.3.28.14). Image credit: Davide Lomeo.

Samples were collected throughout Winam Gulf in a collaborative effort between North American and African participants of the NSF-IRES Advanced Studies Institute on Water Quality and Harmful Algal Blooms in Lake Victoria, Kenya (2022–2023). Sampling conducted aboard the RV *Uvumbuzi* spanned temporal, spatial, biogeochemical, and biotic gradients (Table 1) (Fig. 1). Depth-discrete samples (z = 1 m) were collected *via* Van Dorn sampling in tandem with physiochemical parameters (5). Planktonic biomass was collected onto 0.22 µm pore-size Sterivex filters (Sigma Aldrich) (60–300 mL whole water), fixed with DNA/RNA Shield (Zymo Research), and stored at room temperature until DNA extraction (~3 weeks). Opportunistic samples were collected *via* canoe from Naivasha (July 2022) and *via* shore access from Simbi (June 2022) and riverine systems (May 2023) in the same manner as described above. All 2022 DNA extractions were performed at the University of Tennessee Knoxville using a standard basic phenol chloroform protocol, followed by ethanol precipitation (6, 7). All 2023 DNA extractions were performed at the University of Michigan using the standard protocol of the DNeasy PowerWater Sterivex extraction kit (Qiagen). Samples were assessed for quality *via* Nanodrop (Thermo Fisher Scientific) and quantity *via* Qubit dsDNA HS assay (Invitrogen). Sequencing of 2022 samples was performed at the University of Minnesota Genomics Center (following standard Illumina Nextera XT library preparation) on the NovaSeq S4 (2 × 150 bp paired-end) yielding ~200 million reads per sample. Sequencing of the 2023 samples was performed at the University of Michigan Advanced Genomics Core (following standard Illumina NEBNext Ultra II FS DNA library preparation on the NovaX 10B (2 × 150 bp paired-end) yielding ~200 million reads per sample. Fastq files were preprocessed and quality controlled *via* fastp (v.0.20.0) (8, 9).

**TABLE 1:** Spatial, temporal, and biogeochemical characteristics of metagenomic libraries^*a*^^,*b*^

SRA	Seq ID	Lat /Long	LV site and sample	Date	# Raw reads	[MCY]	%CYA
SRS21505105	LV_1	−0.1961, 34.38861	Asembo Bay-WC-R1	06–25-2022	227353810	0.11	27.26
SRS21505106	LV_2	−0.1961, 34.38861	Asembo Bay-WC-R2	06–25-2022	223816760	0.11	27.26
SRS21505118	LV_3	−0.2331, 34.49639	Ndere Island-WC-R1	06–25-2022	156154078	0.03	60.18*
SRS21505128	LV_4	−0.2331, 34.49639	Ndere Island-WC-R2	06–25-2022	176524208	0.03	60.18*
SRS21505139	LV_5	−0.2466, 34.60327	Mid-Gulf-WC-R1	06–25-2022	212425512	0.14	54.01
SRS21505148	LV_6	−0.2466, 34.60327	Mid-Gulf-WC-R2	06–25-2022	184011628	0.14	54.01
SRS21505150	LV_7	−0.4155, 34.20972	Mbita East-WC-R1	06–24-2022	196658386	0.03	23.41
SRS21505149	LV_8	−0.4155, 34.20972	Mbita East-WC-R2	06–24-2022	185741920	0.03	23.41
SRS21505151	LV_9	−0.4155, 34.20972	Mbita East-PN-R1	06–24-2022	206770084	0.03	23.41
SRS21505152	LV_10	−0.4155, 34.20972	Mbita East-PN-R2	06–24-2022	199028244	0.03	23.41
SRS21505107	LV_11	−0.5216, 34.45703	Homa Bay-WC-R1	06–24-2022	157096488	0.19	62.34*
SRS21505109	LV_12	−0.5216, 34.45703	Homa Bay-WC-R2	06–24-2022	237301584	0.19	62.34*
SRS21505108	LV_13	−0.4525, 34.41326	Soklo-WC-R1	06–24-2022	248531444	0.11	52.31
SRS21505110	LV_14	−0.4525, 34.41326	Soklo-WC-R2	06–24-2022	203748428	0.11	52.31
SRS21505111	LV_15	−0.5220, 34.45441	H.B. Pier-WC-R1	06–24-2022	206992708	1.34*	62.02*
SRS21505113	LV_16	−0.5220, 34.45441	H.B. Pier-WC-R2	06–24-2022	181842672	1.34*	62.02*
SRS21505112	LV_17	−0.5214, 34.45083	H.B. H_2_O Plant-WC-R1	06–24-2022	21812022	0.19	66.40*
SRS21505114	LV_18	−0.5214, 34.45083	H.B. H_2_O Plant-WC-R2	06–24-2022	195838482	0.19	66.40*
SRS21505115	LV_19	−0.5216, 34.45703	H.B. boats-WC-R1	06–24-2022	256680112	0.10	43.16
SRS21505116	LV_20	−0.5216, 34.45703	H.B. boats-WC-R2	06–24-2022	212861604	0.10	43.16
SRS21505117	LV_21	−0.4200, 34.20583	Mbita West-WC-R1	06–24-2022	233461334	NA	35.43
SRS21505119	LV_22	−0.4200, 34.20583	Mbita West-WC-R2	06–24-2022	175707410	NA	35.43
SRS21505120	LV_23	−0.1041, 34.74074	Kisumu-WC-R1	05–29-2023	213172076	0.378	49.95
SRS21505121	LV_24	−0.1539, 34.72810	Dunga-WC-R1	05–29-2023	187393076	0.200	11.62
SRS21505122	LV_25	−0.3185, 34.75242	Sondu Miriu-WC-R1	05–29-2023	203373948	0.363	19.53
SRS21505123	LV_26	−0.2572, 34.70315	Mid-Gulf-WC-R1	05–29-2023	205929498	0.247	29.51
SRS21505124	LV_27	−0.3471, 34.64945	Awach-WC-R1	05–30-2023	198123886	0.079	19.60
SRS21505125	LV_28	−0.3144, 34.56518	Bala Rawi-WC-R1	05–30-2023	205365924	0.150	7.15
SRS21505127	LV_29	−0.3505, 34.45655	Ingra-WC-R1	05–30-2023	184704976	0.266	26.80
SRS21505126	LV_30	−0.4400, 34.47360	Kowuor-WC-R1	05–30-2023	172991040	NA	11.62
SRS21505129	LV_31	−0.4731, 34.49207	Oluch-WC-R1	05–31-2023	184763788	0.086	9.54
SRS21505130	LV_32	−0.4294, 34.36400	Sikli-WC-R1	05–31-2023	179016666	0.149	27.75
SRS21505131	LV_33	−0.4779, 34.35530	Mirunda-WC-R1	05–31-2023	200957854	0.311	33.86
SRS21505132	LV_34	−0.4155, 34.20972	Mbita East-WC-R1	06–01-2023	192116266	0.138	34.44
SRS21505133	LV_35	−0.4233, 34.19795	Mbita West-WC-R1	06–01-2023	203786486	NA	36.44
SRS21505134	LV_36	−0.4055, 34.10967	Bridge Island-WC-R1	06–01-2023	170250064	0.121	86.92
SRS21505135	LV_37	−0.2683, 34.14810	Bondo-WC-R1	06–02-2023	190660000	0.367	NA
SRS21505136	LV_38	−0.0623, 34.03812	Yala-WC-R1	06–02-2023	176826424	0.713	88.96*
SRS21505137	LV_39	−0.3410, 34.2403	Rosinga-WC-R1	06–03-2023	179478494	0.097	37.17
SRS21505138	LV_40	−0.3742, 34.23967	Rosinga-WC-R1	06–03-2023	181580836	0.379	2.95
Opportunistic outgroup samples from adjacent lakes and riverine systems							
SRS21505140	NR_1	−0.1695, 34.9211	Nyando River-WC-R1	05–29-2023	190511542	NA	NA
SRS21505141	SRB_1	−0.3973, 35.01796	Sondu River-WC-R1	05–29-2023	196920444	NA	NA
SRS21505142	SRS_1	−0.3478, 34.79111	Sondu River-WC-R1	05–29-2023	179246390	NA	NA
SRS21505143	KR_1	−0.3800, 34.63508	Kiboun River-WC-R1	05–30-2023	190344778	NA	NA
SRS21505144	LS_1	−0.3697, 34.62892	Lake Simbi-WC-R1	07–02-2022	211554128	NA	NA
SRS21505145	LS_2	−0.3697, 34.62892	Lake Simbi-WC-R2	07–02-2022	223160638	NA	NA
SRS21505146	LN_1	−0.7586, 36.4250	Lake Naivasha-WC-R1	07–12-2022	213941526	NA	NA
SRS21505147	LN_2	−0.7586, 36.4250	Lake Naivasha-WC-R2	07–12-2022	261086438	NA	NA

^*a*^ Note WC = sample was collected directly from lake with no pre-filtering, PN = sample was collected with a plankton net (62 μm). Biological replicates are indicated with R1 or R2 for “replicate 1 or replicate 2”. See BCO-DMO for full details (5).

^*b*^ The total number of raw reads per library (total reads) are listed along with microcystin concentration [MCY] and relative proportion of cyanobacteria present in the water column *vs* the whole photosynthetic community (% CYA). Microcystin concentration at each sample site is noted (μg·L^−1^) with levels above the suggested limit of detection for ELISA kit assays indicated (*). Relative abundance of cyanobacteria (cells·L^−1^) was determined *via* an Algae Torch (bbe Moldaenke, Kiel, Germany) and calculated as follows: % CYA = (# cells with phycocyanin / # cells with chlorophyll *a*) * 100. Sites where cyanobacteria were >55% of total Chl *a* were deemed “blooms” (10) and thus indicated with a (*) ; NA, data not available.

There is a critical need for harmonized monitoring of the African Great Lakes (11). Climatic and anthropogenic changes threaten to exacerbate freshwater cyanobacterial blooms across the globe (12, 13) – with residents along the African Great Lakes at a disproportionately higher risk (1, 3, 4). This work serves as a foundational resource for future molecular studies concerning Lake Victoria and its surrounding watershed.

## Data Availability

Raw sequences are available on the NCBI SRA under BioProject PRJNA1110566 and BioSamples SAMN41659670-SAMN41659717. Biogeochemical data are available on the Biological and Chemical Oceanography Data Management Office (BCO-DMO) (5).
